# Pre-Dialysis Trajectory of Brain Natriuretic Peptide Levels and Body Weight in Chronic Kidney Disease Patients: A Predictive Marker for Unplanned Dialysis Initiation

**DOI:** 10.1016/j.xkme.2026.101349

**Published:** 2026-04-01

**Authors:** Shinnosuke Sugihara, Takashin Nakayama, Tatsuhiko Azegami, Kaori Hayashi

**Affiliations:** Division of Nephrology, Endocrinology, and Metabolism, Department of Internal Medicine, Keio University School of Medicine, Tokyo, Japan

**Keywords:** Brain natriuretic peptide, body weight, dialysis, kidney failure, risk factors

## Abstract

**Rationale & Objective:**

Unplanned dialysis initiation in chronic kidney disease (CKD) patients is associated with adverse outcomes, yet reliable predictors for optimal dialysis timing remain elusive. The clinical significance of longitudinal changes in brain natriuretic peptide (BNP) levels and body weight (BW) in this context is unclear. We examined whether these parameters predict the risk of unplanned dialysis.

**Study Design, Setting & Population:**

This retrospective cohort study included 231 CKD patients initiating maintenance dialysis between 2014 and 2024 at a single Japanese university hospital.

**Predictors & Outcomes:**

Predictors were BNP levels and BW ratios, defined as the 3-month value divided by the average of values obtained 6-12 months before dialysis initiation. The primary outcome was unplanned dialysis initiation.

**Analytical Approach:**

We assessed associations between BNP levels or BW ratio and unplanned dialysis initiation using multivariable logistic regression models.

**Results:**

Among 231 patients included in the analysis, 137 (59%) had planned and 94 (41%) unplanned dialysis initiation. The median (interquartile range) age was 70 (61-79) years, and 67 (29%) were female. BNP levels increased toward dialysis initiation, particularly in the unplanned group, whereas BW remained largely stable in both groups. A high BNP ratio (>median) was significantly associated with an increased likelihood of unplanned dialysis initiation (odds ratio [OR], 5.64; 95% confidence interval [CI], 2.90-10.99). For the BW ratio, both weight loss (<25th percentile; OR, 3.47; 95% CI, 1.58-7.65) and weight gain (>75th percentile; OR, 2.33; 95% CI, 1.11-4.87) were associated with an increased likelihood of unplanned dialysis initiation.

**Limitations:**

Retrospective, single-center design with possible residual confounding and limited generalizability; unavailability of body composition data.

**Conclusions:**

In patients with advanced CKD, trajectories of BNP, together with BW, were significant predictors of unplanned dialysis initiation. Close monitoring of these parameters might help identify high-risk patients and facilitate a timely dialysis transition.

## Plain-Language Summary

For people with advanced chronic kidney disease, timely dialysis initiation is important for better outcomes. However, there is still not enough evidence to determine the optimal time to start dialysis, which makes this goal difficult to achieve. Focusing on fluid overload—a common reason for dialysis—we examined the potential of 2 fluid status markers, brain natriuretic peptide (BNP) levels and body weight (BW), as predictors of unplanned dialysis initiation. Among 231 patients starting dialysis, a marked increase in pre-dialysis BNP levels was associated with a higher incidence of unplanned dialysis, whereas BW loss and gain were similarly linked to increased risk. These findings suggest that monitoring BNP levels and BW trends might help inform the timing of dialysis initiation.

Chronic kidney disease (CKD) is a global health burden, affecting approximately 10% of the adult population.^1^ As CKD progresses to kidney failure, kidney replacement therapy becomes necessary.^2^ However, the optimal timing for initiating dialysis remains a matter of debate, as reflected by substantial variation in estimated glomerular filtration rate (eGFR) at the time of dialysis initiation. Previous studies have shown that a considerable proportion of patients initiate dialysis in an unplanned manner.^3^^,^^4^ Importantly, such unplanned dialysis initiation is associated with unfavorable clinical outcomes, including prolonged hospital stay and increased mortality, when compared with planned initiation.^5^^,^^6^ Therefore, identifying strategies to facilitate timely and appropriate dialysis initiation is an important area of current research.

Disorders of fluid balance remain among the most complex and clinically significant challenges in the management of kidney failure. Indeed, fluid overload is one of the leading causes of unplanned dialysis initiation. In this study, we focused on brain natriuretic peptide (BNP), a simple and sensitive biomarker secreted by the ventricles in response to volume or pressure overload. BNP is widely used in the clinical management of heart failure.^7^^,^^8^ However, its typical trajectory in CKD patients has not been fully characterized, and its value in guiding decisions regarding dialysis initiation remains uncertain.

Accordingly, this retrospective study primarily investigated the trajectory of BNP levels during the pre-dialysis period and its association with the risk of unplanned dialysis initiation. Body weight (BW), a traditional marker of volume status, was additionally assessed as a supportive reference parameter.

## Materials and Methods

### Study Participants

The present study included patients who started maintenance dialysis at Keio University Hospital between April 2014 and March 2024. Patients were excluded if BNP levels were unavailable at any of the following time points: within 6-12 months, at 3 months, or at dialysis initiation. Additionally, patients who were using angiotensin receptor-neprilysin inhibitors (ARNIs), which could influence BNP levels, or those who had undergone kidney transplantation were excluded. All procedures involving human participants were conducted in accordance with the ethical standards of the institutional and/or national research committee and in accordance with the 1964 Helsinki Declaration and its subsequent amendments or comparable ethical standards. The study protocol was reviewed and approved by the Ethics Committee of Keio University School of Medicine (approval number: 20241197). Informed consent was obtained via an opt-out approach provided on the website.

### Data Collection and Measurements

BNP values were obtained at 5 time points: 12 months, 9 months, 6 months, and 3 months before dialysis initiation as well as at the time of initiation. Baseline BNP was defined as the mean of values obtained between 6 and 12 months before dialysis initiation (ie, a single value if only 1 measurement was available; the arithmetic mean if 2 or 3 were available). The BNP ratio was calculated by dividing the BNP value at 3 months before dialysis by the baseline BNP value. The 3-month time point was chosen to ensure an adequate and safe preparation period for a smooth transition to dialysis, as part of a predictive approach. Baseline and ratio values of BW and eGFR were calculated in the same manner. To account for substantial interindividual variability, the ratio of follow-up to baseline values was used for both BNP and BW in this study. In addition, laboratory parameters, including serum creatinine (mg/dL), urea nitrogen (mg/dL), potassium (mEq/L), and hemoglobin (g/dL) levels, were collected at the same 5 time points. The eGFR was determined using the formula: 194 × Cr^-1.094^ × Age^-0.287^ (×0.739 if female).^9^ Demographic and clinical characteristics were collected at 3 months before dialysis initiation. These characteristics included age, sex, smoking and alcohol use history, height, blood pressure measured at our institution, comorbid conditions (hypertension, diabetes mellitus, heart failure, coronary artery disease, cerebrovascular disease, and malignancy), and medications (renin–angiotensin system [RAS] inhibitors, β-blockers, loop diuretics, potassium binders, erythropoiesis-stimulating agents, and statins). Comorbid conditions, including hypertension, were determined based on documented past medical history in the patient’s medical records.

### Unplanned Dialysis Initiation as the Primary Outcome

At our institution, dialysis is typically initiated during hospitalization irrespective of patient background or clinical symptoms. Based on previous studies, unplanned dialysis initiation was defined as any of the following: (i) the use of an uncuffed dialysis catheter; (ii) an emergency hospitalization specifically for the purpose of initiating dialysis; or (iii) unscheduled initiation of dialysis during hospitalization for other reasons. All other cases were classified as planned dialysis initiation.^10^ To ensure consistency and reliability, the classification of dialysis initiation (planned vs unplanned)—the primary outcome of this study—was determined by consensus between 2 investigators (SS and TN). The main clinical indication for starting dialysis was classified into 1 of 4 mutually exclusive categories: volume overload, uremia, hyperkalemia, and acidosis. Each patient was assigned to a single category based on the predominant indication. In addition, we assessed how the pattern of dialysis initiation (planned vs unplanned) was associated with clinical outcomes, specifically the length of hospital stay and the initial dialysis modality, either hemodialysis (HD) or peritoneal dialysis (PD).

### Statistical Analyses

Continuous variables are presented as medians with interquartile ranges (25th-75th percentiles), and categorical variables are presented as percentages. Patients were categorized into 2 groups based on the median BNP ratio (low or high BNP increment). In addition, patients were categorized into three groups according to BW ratio: below the 25th percentile (weight loss), between the 25th and 75th percentiles (weight stable), and above the 75th percentile (weight gain). The BNP ratio was assumed to have a linear association with unplanned dialysis initiation, whereas the BW ratio was categorized into 3 groups based on quartiles to account for its potentially nonlinear relationship given that changes in BW may reflect either fluid overload or poor nutritional status related to uremia. Differences in continuous variables between groups were examined using the *t* test or Mann–Whitney *U* test for 2-group comparisons and one-way analysis of variance or Kruskal–Wallis test for 3-group comparisons, as appropriate based on data distribution. Categorical variables were compared using the chi-square test. The association between BNP and BW ratios—both considered markers for volume status—was visually assessed using a scatter plot with locally estimated scatterplot smoothing (LOESS). In addition, we performed a similar analysis comparing these markers with eGFR ratio.

A restricted cubic spline analysis with 3 knots (at the 10th, 50th, and 90th percentiles) was performed to assess the linearity assumption between the BNP ratio and the risk of unplanned dialysis initiation.^11^ Confounders included age, sex, mean blood pressure, Charlson comorbidity index (CCI), potassium level, eGFR, and the use of RAS inhibitors, with the median value serving as the reference point. Subsequently, logistic regression model adjusted for the same variables was employed to estimate odds ratios (ORs) with 95% confidence intervals (CIs). This approach was selected because all patients eventually initiated dialysis, and the outcome of interest was the type of initiation (planned vs unplanned), rather than its timing. The same analysis was also applied to BW ratio.

We conducted 3 sensitivity analyses to validate the robustness of our findings. First, recognizing that the clinical context of dialysis initiation varies across settings, we performed a sensitivity analysis redefining unplanned initiation as the use of uncuffed catheters. Second, given the well-established role of BNP as a marker for heart failure, we substituted heart failure for the CCI as an independent variable in the multivariable model. Third, multiple imputation by chained equations (30 imputations) was performed to address missing values for BNP and BW; no missing data were present in other covariates.

All analyses were performed using STATA version 18.0 (StataCorp LLC, College Station, TX, USA), and figures were created using GraphPad Prism version 9 (GraphPad Software, San Diego, CA, USA). A *P* value of less than 0.05 was considered statistically significant.

## Results

### Patient Characteristics

**Figure 1:**
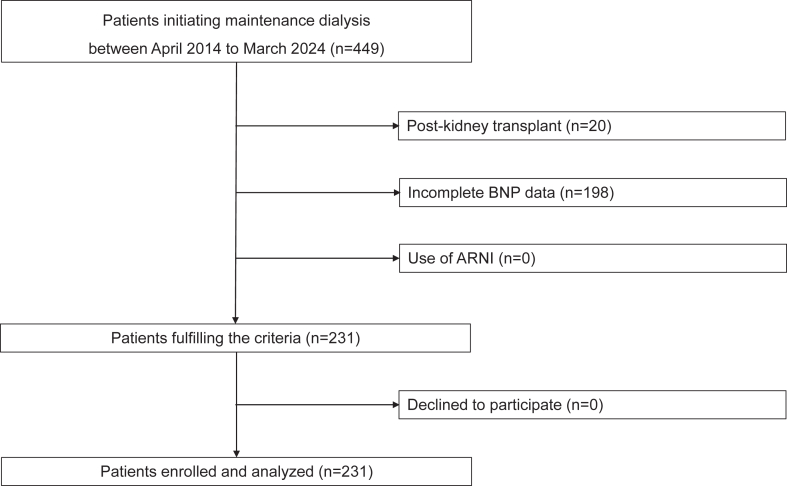
Flow diagram of patient selection. Among 449 patients initiating maintenance dialysis between April 2014 and March 2024, 218 were excluded because of previous kidney transplantation (n = 20), incomplete brain natriuretic peptide (BNP) data (n = 198), or use of angiotensin receptor-neprilysin inhibitors (ARNI) (n = 0). Thus, 231 patients were included in the final analysis.

Among 449 patients who initiated maintenance dialysis, 20 were excluded as a result of postkidney transplant, 198 because of incomplete BNP data, and none because of ARNI use, resulting in a final cohort of 231 patients (Fig 1). Table 1 summarizes the characteristics of patients at 3 months before dialysis initiation. The median age was 70 (61-79) years, with 67 (29%) being female. Diabetic kidney disease (DKD) was the most common underlying cause of CKD, affecting 90 patients (40%). Heart failure was present in 69 patients (30%), and 82 (36%) were receiving RAS inhibitors. Patient characteristics stratified by the median BNP ratio (≤1.5 vs >1.5) are presented in Table S1. Compared with the low BNP increment group, the high BNP increment group exhibited higher mean blood pressure, a greater prevalence of DKD and nephrosclerosis as underlying causes of CKD, and a higher CCI score. Table S2 presents the clinical features of patients divided into 3 groups according to the BW ratio: the lowest quartile (≤0.95), interquartile range (0.95-1.00), and highest quartile (≥1.00). No significant differences were observed among the 3 groups across all variables. The 198 excluded patients because of missing BNP data had median age 70 (55-79) years. Moreover, 60 (30%) were female, 84 (42%) had diabetes mellitus, and 43 (22%) had heart failure. There were no significant baseline differences between the two groups, except that heart failure was marginally less frequent among the excluded patients.

**Table 1:** Clinical Background of Participants 3 Months Before Dialysis Initiation.

Variables	Total (n = 231)
Age, y	70 (61-79)
Sex (female)	67 (29%)
Body mass index, kg/m^2^	23 (20-26)
Smoking history	134 (58%)
Alcohol use history	97 (42%)
Systolic blood pressure, mm Hg	145 (127-157)
Diastolic blood pressure, mm Hg	76 (65-87)
Mean blood pressure, mm Hg	99 (88-108)
Causes of chronic kidney disease	
Diabetic kidney disease	90 (40%)
Nephrosclerosis	50 (22%)
Chronic glomerulonephritis	40 (17%)
Others	51 (22%)
Comorbid conditions	
Hypertension	215 (93%)
Diabetes mellitus	112 (48%)
Coronary artery disease	46 (20%)
Heart failure	69 (30%)
Malignancy	32 (14%)
Charlson comorbidity index	4 (3-4)
Medications	
Renin–angiotensin system inhibitors	82 (36%)
Beta blockers	73 (32%)
Loop diuretics	135 (58%)
Potassium binders	59 (26%)
Erythropoiesis-stimulating agent	214 (93%)
Statins	83 (36%)

Figure S1 presents a scatter plot illustrating the relationship between BNP and BW ratios, with LOESS analysis suggesting an absence of meaningful correlation. A similar lack of association was observed between these values and eGFR ratios.

### Dialysis Initiation Patterns and Changes in Clinical Data During the Pre-dialysis Phase

A total of 94 patients (41%) initiated maintenance dialysis in an unplanned manner, among whom 64 (68%) received an uncuffed dialysis catheter (Table 2). Compared with those with planned initiation, patients with unplanned initiation had a longer hospital stay (10 vs 29 days, *P* < 0.01) and were less likely to undergo PD as their initial kidney replacement therapy (23% vs 1%, *P* < 0.01). The predominant indication for dialysis initiation differed by group. Specifically, volume overload was most common in the unplanned group (60 patients [64%]), whereas uremia was the leading cause in the planned group (103 patients [74%]).

**Table 2:** Differences Between Planned Versus Unplanned Dialysis Initiation

	Planned dialysis (n = 137)	Unplanned Dialysis (n = 94)	*P* Value
Hospital days	10 (6-14)	29 (19-47)	<0.01
Use of uncuffed catheter	0 (0%)	64 (68%)	<0.01
Dialysis modality			<0.01
Hemodialysis	105 (77%)	93 (99%)	
Peritoneal dialysis	32 (23%)	1 (1%)	
Primary reasons for dialysis initiation			<0.01
Volume overload	31 (23%)	60 (64%)	
Uremia	103 (74%)	28 (30%)	
Hyperkalemia	3 (2%)	3 (3%)	
Acidosis	0 (0%)	3 (3%)	

**Figure 2:**
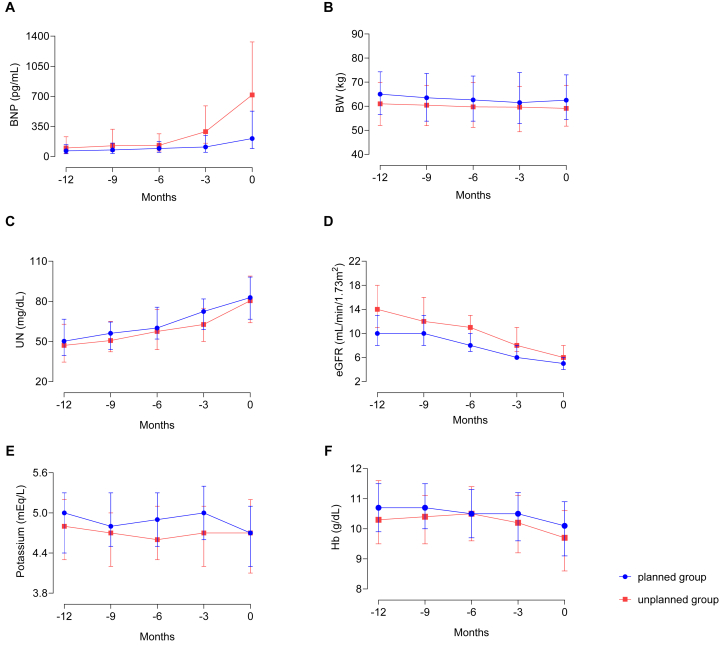
Longitudinal changes in clinical parameters during the 12 months preceding dialysis initiation, stratified by initiation pattern. Trajectories for (A) brain natriuretic peptide (BNP), (B) body weight (BW), (C) urea nitrogen (UN), (D) estimated glomerular filtration rate (eGFR), (E) serum potassium level, and (F) hemoglobin (Hb) level are shown for patients with planned (•) and unplanned (▪) dialysis initiation. Values are presented as median with interquartile range.

Figure 2 illustrates the 1-year changes of clinical parameters preceding dialysis initiation according to initiation patterns (detailed values in Table S3). Overall, BNP levels increased gradually until 6 months before dialysis initiation, after which they increased sharply in the unplanned group. In contrast, an upward trend was observed in the planned group from 3 months prior. BW remained largely stable in both the planned and unplanned groups, with no significant differences in their trajectories. There were no notable differences in the remaining parameters, except for a more rapid decline in eGFR among patients in the unplanned group.

### Associations of BNP and BW Changes with Unplanned Dialysis Initiation

**Figure 3:**
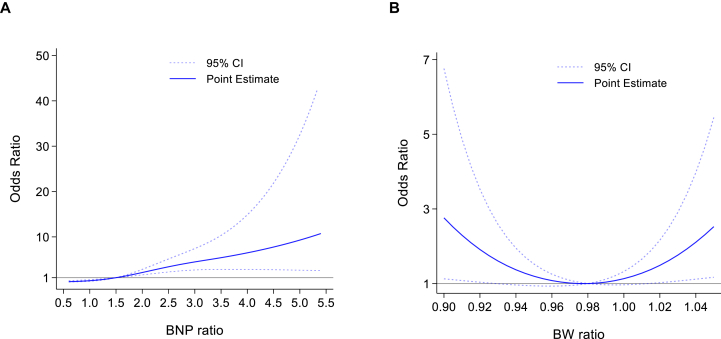
Association between biomarker ratios and unplanned dialysis initiation assessed by adjusted restricted cubic spline analysis. (A) Brain natriuretic peptide (BNP) ratio. (B) Body weight (BW) ratio. Solid lines represent the adjusted odds ratio (OR), and dotted lines represent the 95% confidence intervals (CI). The reference point (OR = 1) is set at the median value of each respective ratio. Models were adjusted for age, sex, Charlson comorbidity index, potassium level, estimated glomerular filtration rate, and use of renin–angiotensin system inhibitors.

The results of the restricted cubic spline analysis are shown in Figure 3. The BNP ratio demonstrated an approximately linear association with the risk of unplanned dialysis initiation, whereas the BW ratio showed a U-shaped relationship. In the logistic regression model, a high BNP increment was significantly associated with an increased risk of unplanned dialysis initiation (OR, 5.64; 95% CI, 2.90-10.99; Table 3). Regarding the BW ratio, both weight loss and weight gain were significantly associated with unplanned dialysis initiation compared with weight stability, with ORs of 3.47 (95% CI, 1.58-7.65) and 2.33 (95% CI, 1.11-4.87), respectively.

**Table 3:** Results of Logistic Regression Analysis on BNP and BW Changes Associated With Unplanned Dialysis Initiation

	Model 1	Model 2	Model 3
BNP			
Low increment	1 [Reference]	1 [Reference]	1 [Reference]
High increment	5.64 (3.16-10.08)	5.63 (3.11-10.21)	5.64 (2.90-10.99)
BW			
Weight stable	1 [Reference]	1 [Reference]	1 [Reference]
Weight loss	3.53 (1.79-6.94)	3.57 (1.77-7.19)	3.47 (1.58-7.65)
Weight gain	1.97 (1.02-3.80)	2.04 (1.04-3.99)	2.33 (1.11-4.87)

*Notes*: BNP analyses were conducted in all 231 participants, whereas analyses of BW change were performed in 225 participants after excluding six with missing data. Odds ratios with 95% confidence interval are presented. Model 1 is unadjusted. Model 2 is adjusted for age and sex. Model 3 is adjusted for age, sex, mean blood pressure, Charlson comorbidity index, potassium level, estimated glomerular filtration rate, and use of renin–angiotensin system inhibitors.

Abbreviations: BW, body weight; BNP, B-type natriuretic peptide.

We performed 3 sensitivity analyses. First, we redefined unplanned dialysis initiation solely based on the use of an uncuffed dialysis catheter (Table 4). In this analysis (64 events), a high BNP ratio remained a significant predictor of unplanned dialysis initiation (OR, 3.06; 95% CI, 1.53-6.13), and similar trends were observed for both weight loss (OR, 3.66; 95% CI, 1.61-8.31) and weight gain (OR, 2.18; 95% CI, 0.97-4.90). Second, we replaced the CCI with heart failure as a covariate in the multivariable mode (Table S4). This analysis showed that a high BNP increment (OR, 6.72; 95% CI, 3.42-13.23) as well as weight fluctuations (OR, 3.71; 95% CI, 1.70-8.07 for weight loss; OR, 2.27; 95% CI, 1.10-4.70 for weight gain) were related to an increased risk of unplanned dialysis initiation. Third, we performed multiple imputations for missing data, confirming that our primary findings remained unchanged (OR, 5.30; 95% CI, 2.63–10.68 for high BNP increment; OR, 3.52; 95% CI, 1.59-7.78 for weight loss; OR, 2.42; 95% CI, 1.18;4.98 for weight gain; Table S5).

**Table 4:** Results of Logistic Regression Analysis on BNP and BW Changes Associated With Unplanned Dialysis Initiation (Defined by the Use of Uncuffed Catheters)

	Model 1	Model 2	Model 3
BNP			
Low increment	1 [Reference]	1 [Reference]	1 [Reference]
High increment	3.36 (1.81-6.24)	3.26 (1.74-6.11)	3.06 (1.53-6.13)
BW			
Weight stable	1 [Reference]	1 [Reference]	1 [Reference]
Weight loss	3.50 (1.71-7.17)	3.74 (1.78-7.84)	3.66 (1.61-8.31)
Weight gain	1.82 (0.87-3.80)	1.88 (0.89-3.98)	2.18 (0.97-4.90)

*Notes*: BNP analyses were conducted in all 231 participants, whereas analyses of BW change were performed in 225 participants after excluding six with missing data. Odds ratios with 95% confidence interval are presented. Model 1 is unadjusted. Model 2 is adjusted for age and sex. Model 3 is adjusted for age, sex, mean blood pressure, Charlson comorbidity index, potassium level, estimated glomerular filtration rate, and use of renin–angiotensin system inhibitors.

Abbreviations: BNP, B-type natriuretic peptide; BW, body weight.

## Discussion

In this retrospective cohort study of 231 patients initiating maintenance dialysis, we found that an increase in BNP levels and both BW loss and gain during the year preceding dialysis initiation were significantly associated with the risk of unplanned dialysis initiation. These associations remained consistent across multiple sensitivity analyses, supporting the robustness of our findings.

Although BNP level is one of the key biomarkers for assessing volume status and disease progression in patients with heart failure, evidence supporting its use in the CKD population remains limited.^12^ Oka et al^13^ recently demonstrated that BNP monitoring among patients with CKD was associated with a reduce incidence of acute kidney injury, presumably because of improved fluid status control. As CKD progresses, fluid retention tends to worsen because of declining glomerular filtration rate and impaired cardiac function.^14^ In fact, volume overload is a primary driver of dialysis initiation, particularly in emergency settings.^15^ Taken together, BNP may serve as a promising indicator for dialysis initiation. It should be noted that there is substantial interindividual variability in BNP levels, primarily because of differences in underlying cardiac conditions.^16^ To address this, we conducted a longitudinal analysis of BNP levels in pre-dialysis CKD patients to clarify its clinical relevance. Although BW is another classical indicator of fluid status, data on its trajectory during the pre-dialysis phase of kidney failure remain scarce.^17^ This knowledge gap prompted us to evaluate its potential significance in this context.

In this study, BNP levels increased gradually until several months before dialysis initiation, after which they increased sharply. As anticipated, the high BNP ratio was a significant predictor of unplanned dialysis initiation, demonstrating an approximately linear association. This finding is plausible given that volume overload was the predominant clinical indication for dialysis initiation. In contrast, BW remained generally stable during the pre-dialysis period. Interestingly, fluctuations in BW showed a U-shaped association with unplanned dialysis initiation. Although the underlying mechanisms remain uncertain, several possibilities may account for this observation. Weight loss in advanced CKD often reflects poor nutritional status or progressive muscle wasting, both of which may accelerate the onset of uremic symptoms and lead to emergent dialysis.^18^ On the other hand, weight gain is likely indicative of overt volume overload, potentially leading to more frequent events through cardiorenal complications. Indeed, previous studies have reported that, in patients with CKD, not only obesity and underweight but also abrupt changes in BW are associated with subsequent unfavorable outcomes.^19^, ^20^, ^21^

The present study has significant clinical implications. The Kidney Disease: Improving Global Outcomes guidelines recommend that decisions regarding dialysis initiation should be based not on a fixed eGFR threshold but on a comprehensive assessment of symptoms, signs, and laboratory abnormalities.^22^ Among these factors, fluid status remains particularly challenging to evaluate. It is noteworthy that patients in the unplanned group had higher eGFR values at the beginning of follow-up, possibly reflecting a state of “deceptive stability” maintained by chronic fluid overload. This observation could underscore the potential imprecision of relying solely on eGFR to assess kidney function in advanced CKD. Our findings suggest that BNP assessment in this population may provide a valuable tool to support a patient-centered approach—either by optimizing diuretic therapy or, in the setting of diuretic resistance, by identifying the appropriate timing for dialysis initiation. In addition, the absence of an evident association between BNP and BW changes may serve as a caution against over-reliance on BW for fluid assessment in advanced-stage CKD. During the pre-dialysis phase, patients are particularly susceptible to malnutrition attributable to uremia and dietary protein restriction, making it difficult to interpret weight fluctuations accurately.^23^^,^^24^ Therefore, a comprehensive strategy that includes BNP measurements, along with conventional methods such as BW monitoring, physical examination, and chest radiography, could be more effective for fluid assessment in this population.

We acknowledge several limitations in this study. Its observational design raises the possibility of residual confounding despite adjustment for multiple covariates. For example, data on residual diuresis (remaining urine output)—one of the key determinants of volume status associated with BNP and body weight—were unavailable, which represents an important limitation. Information bias may also have occurred because of the retrospective nature of the study; however, 2 investigators independently reviewed unplanned dialysis classification to minimize misclassification. Importantly, although we demonstrated that an increase in BNP levels 3 months before dialysis initiation predicted the occurrence of unplanned dialysis, this finding does not necessarily imply that routine BNP monitoring would reduce the risk. A prospective interventional study to verify this relationship represents an intriguing area for future research. In this study, approximately half of the patients who initiated maintenance dialysis were excluded, primarily because of missing BNP data. Although no apparent differences were observed within the scope of available data, concerns regarding potential selection bias remain. This study was conducted at a single university hospital. Although the eGFR at dialysis initiation was broadly consistent with the Japanese average, dialysis practices, including the timing of vascular access creation, vary considerably across countries and regions.^25^^,^^26^ Of note, RAS inhibitor use appeared to be relatively low in this cohort, partly reflecting the inclusion of patients treated before the evidence for their role in advanced CKD had accumulated.^22^^,^^27^ Therefore, caution is warranted when extrapolating these findings to contemporary populations in other settings. Over the past few years, ARNIs have gained attention in the management of heart failure. Because ARNIs are known to increase BNP levels, we excluded patients receiving these agents.^28^ With the growing use of ARNIs, this issue should be carefully acknowledged. We were unable to evaluate specific components such as fat and muscle mass because dual-energy X-ray absorptiometry or bioelectrical impedance analysis is not routinely performed at our institution. However, these modalities may be useful for precisely evaluating discrepancies between BNP levels and BW changes, and their respective associations with unplanned dialysis initiation also represent an intriguing subject for future research. In addition, inferior vena cava diameter could be another parameter worth investigating in this context.

In summary, this study identified BNP elevation as a significant predictor of unplanned dialysis initiation, with BW fluctuations also showing a meaningful association. These findings highlight the potential clinical value of serial BNP monitoring, complemented by careful weight assessment, for evaluating volume status in patients with advanced CKD.
