# Supplementary material for: Pre-Dialysis Trajectory of Brain Natriuretic Peptide Levels and Body Weight in Chronic Kidney Disease Patients: A Predictive Marker for Unplanned Dialysis Initiation
Source: Kidney Med. 2026 Apr 1;8(6):101349. doi: 10.1016/j.xkme.2026.101349 (PMC13158758; doi:10.1016/j.xkme.2026.101349)

Table S1. Clinical background of participants three months before dialysis initiation divided in two groups based on median BNP increment

Variables	Low BNP increment (n=117)	High BNP increment (n=114)	P value
Age, year	70 (61–78)	72 (61–80)	0.24
Sex (Female)	36 (31%)	31 (27%)	0.55
Body Mass Index, kg/m^2	23 (20–26)	23 (20–25)	0.88
Smoking history	71 (61%)	63 (55%)	0.40
Alcohol use history	49 (42%)	48 (42%)	0.97
Systolic blood pressure, mmHg	141 (126–153)	150 (131–162)	0.09
Diastolic blood pressure, mmHg	76 (64–84)	78 (65–89)	0.16
Mean blood pressure, mmHg	97 (88–106)	102 (89–110)	0.03
Causes of chronic kidney disease			
Diabetic kidney disease	39 (33%)	51 (45%)	0.02
Nephrosclerosis	21 (18%)	29 (25%)	
Chronic glomerulonephritis	23 (20%)	17 (15%)	
Others	34 (29%)	17 (15%)	
Comorbidities			
Hypertension	107 (91%)	108 (95%)	0.33
Diabetes mellitus	54 (46%)	58 (51%)	0.47
Coronary artery disease	22 (19%)	24 (21%)	0.67
Heart failure	30 (26%)	39 (34%)	0.16
Malignancy	13 (11%)	19 (17%)	0.22
Charlson comorbidity index	3 (2–4)	4 (3–5)	0.002
Medications			

Renin–angiotensin system inhibitors	42 (36%)	40 (35%)	0.90
Beta blockers	37 (32%)	36 (32%)	0.99
Loop diuretics	63 (54%)	72 (63%)	0.15
Potassium binders	30 (26%)	29 (25%)	0.97
Erythropoiesis–stimulating agent	109 (93%)	105 (92%)	0.76
Statins	44 (38%)	39 (34%)	0.59

Study participants were categorized into three groups by median BNP increment: <1.5 and >1.5.
Abbreviations: BNP, B-type natriuretic peptide.

Table S2. Clinical background of participants three months before dialysis initiation divided into three groups based on body weight changes: the lowest quartile, the interquartile, and the highest quartile

Variables	Weight loss (n=54)	Weight stable (n=113)	Weight gain (n=58)	P value
Age, year	75 (60–82)	68 (61–79)	71 (59–79)	0.37
Sex (Female)	19 (35%)	29 (26%)	17 (29%)	0.45
Body Mass Index, kg/m^2	22 (19–24)	23 (21–27)	23 (21–27)	0.007
Smoking history	26 (48%)	66 (58%)	37 (64%)	0.23
Alcohol use history	22 (41%)	49 (43%)	22 (38%)	0.79
Systolic blood pressure, mmHg	143 (127–156)	147 (125–157)	146 (131–156)	0.76
Diastolic blood pressure, mmHg	79 (66–88)	72 (60–84)	75 (65–84)	0.28
Mean blood pressure, mmHg	97 (83–117)	100 (89–108)	99 (90–107)	0.25
Causes of chronic kidney disease				
Diabetic kidney disease	25 (46%)	38 (34%)	25 (43%)	0.66
Nephrosclerosis	10 (19%)	28 (25%)	10 (17%)	
Chronic glomerulonephritis	8 (15%)	20 (18%)	12 (21%)	
Others	11 (20%)	27 (24%)	11 (19%)	
Comorbidities				
Hypertension	52 (96%)	105 (93%)	53 (91%)	0.56
Diabetes mellitus	28 (52%)	49 (43%)	32 (55%)	0.29
Coronary artery disease	11 (20%)	25 (22%)	10 (17%)	0.76
Heart failure	21 (39%)	28 (25%)	17 (29%)	0.17
Malignancy	6 (11%)	14 (12%)	10 (17%)	0.58
Charlson comorbidity index	4 (3–5)	3 (2–4)	4 (3–4)	0.10
Medications				

Renin–angiotensin system inhibitors	19 (35%)	43 (38%)	16 (28%)	0.39
Beta blockers	17 (31%)	33 (29%)	20 (34%)	0.78
Loop diuretics	37 (69%)	61 (54%)	34 (59%)	0.20
Potassium binders	11 (20%)	36 (32%)	11 (19%)	0.11
Erythropoiesis–stimulating agent	49 (91%)	105 (93%)	54 (93%)	0.86
Statins	16 (30%)	45 (40%)	19 (33%)	0.38

Excluding six participants with missing information on body weight change. The remaining participants were categorized into three groups according to body weight change: <0.95, 0.95–1.00, and >1.00.

Table S3. One-year progression of clinical data prior to dialysis initiation

	12 months ago	9 months ago	6 months ago	3 months ago	At dialysis initiation
BNP, pg/mL	n=185	n=205	n=226	n=231	n=231
Planned	65 (32–138)	76 (35–156)	93 (48–174)	111 (49–242)	209 (92–527)
Unplanned	99 (37–229)	125 (57–317)	130 (63–264)	289 (154–589)	716 (205–1333)
BW, kg	n=196	n=204	n=218	n=225	n=231
Planned	64 (57–74)	64 (54–74)	63 (54–73)	62 (53–74)	63 (55–73)
Unplanned	61 (52–70)	60 (52–69)	60 (51–70)	60 (49–68)	59 (52–69)
UN, mg/dL	n=187	n=207	n=228	n=231	n=231
Planned	50 (40–67)	56 (44–65)	60 (52–76)	72 (59–82)	83 (67–98)
Unplanned	47 (35–63)	51 (42–65)	58 (44–74)	63 (50–75)	81 (64–99)
eGFR, mL/min/1.73m2	n=188	n=207	n=228	n=231	n=231
Planned	10 (8–13)	10 (8–13)	8 (7–10)	6 (6–8)	5 (4–6)
Unplanned	14 (11–18)	12 (10–16)	11 (8–13)	8 (7–11)	6 (5–8)
Potassium level, mEq/L	n=188	n=207	n=228	n=231	n=231
Planned	5.0 (4.4–5.3)	4.8 (4.5–5.3)	4.9 (4.5–5.3)	5.0 (4.6–5.4)	4.7 (4.2–5.1)
Unplanned	4.8 (4.3–5.2)	4.7 (4.2–5.0)	4.6 (4.3–5.1)	4.7 (4.2–5.1)	4.7 (4.1–5.2)
Hb, g/dL	n=188	n=207	n=227	n=231	n=231
Planned	11 (10–12)	11 (10–12)	11 (10–11)	11 (10–11)	10 (9–11)
Unplanned	10 (10–12)	10 (10–11)	11 (10–11)	10 (9–11)	10 (9–11)

Abbreviations: BNP, B-type natriuretic peptide; BW, body weight; UN, urea nitrogen; eGFR, estimated glomerular filtration rate.

Table S4. Results of logistic regression analysis on BNP and BW Changes associated with unplanned dialysis initiation (heart failure as a covariate instead of Charlson comorbidity index)

	Model 1	Model 2	Model 3
BNP			
low increment	1 [Reference]	1 [Reference]	1 [Reference]
high increment	5.64 (3.16–10.08)	5.63 (3.11–10.21)	6.72 (3.42–13.23)
BW			
weight stable	1 [Reference]	1 [Reference]	1 [Reference]
weight loss	3.53 (1.79–6.94)	3.57 (1.77–7.19)	3.71 (1.70–8.07)
weight gain	1.97 (1.02–3.80)	2.04 (1.04–3.99)	2.27 (1.10–4.70)

BNP analyses were conducted in all 231 participants, whereas analyses of BW change were performed in 225 participants after excluding six with missing data. Odds ratios with 95% confidence interval are presented. Model 1 is unadjusted. Model 2 is adjusted for age and sex. Model 3 is adjusted for age, sex, mean blood pressure, heart failure, potassium level, estimated glomerular filtration rate, and use of renin–angiotensin system inhibitors. Abbreviations: BNP, B-type natriuretic peptide; BW, body weight.

Table S5. Results of logistic regression analysis on BNP and BW Changes associated with unplanned dialysis initiation (addressing missing values through multiple imputations)

	Model 1	Model 2	Model 3
BNP			
low increment	1 [Reference]	1 [Reference]	1 [Reference]
high increment	5.27 (2.83–9.81)	5.01 (2.64–9.54)	5.30 (2.63–10.68)
BW			
weight stable	1 [Reference]	1 [Reference]	1 [Reference]
weight loss	3.53 (1.79–6.94)	3.56 (1.76–7.20)	3.52 (1.59–7.78)
weight gain	2.01 (1.06–3.80)	2.09 (1.08–4.01)	2.42 (1.18–4.98)

Odds ratios with 95% confidence interval are presented. Model 1 is unadjusted. Model 2 is adjusted for age and sex. Model 3 is adjusted for age, sex, mean blood pressure, Charlson comorbidity Index, potassium level, estimated glomerular filtration rate, and use of renin–angiotensin system inhibitors. Multiple imputation was applied to address missing values in BNP (at 6, 9, or 12 months before dialysis) and body weight (at 3, 6, 9, or 12 months before dialysis). Abbreviations: BNP, B-type natriuretic peptide; BW, body weight.

Fig S1. Scatter plot illustrating the relationships among the Brain Natriuretic Peptide (BNP) ratio, the Body Weight (BW) ratio, and the estimated Glomerular Filtration Rate (eGFR) ratio. The solid line represents the locally estimated scatterplot smoothing (LOESS) curve.

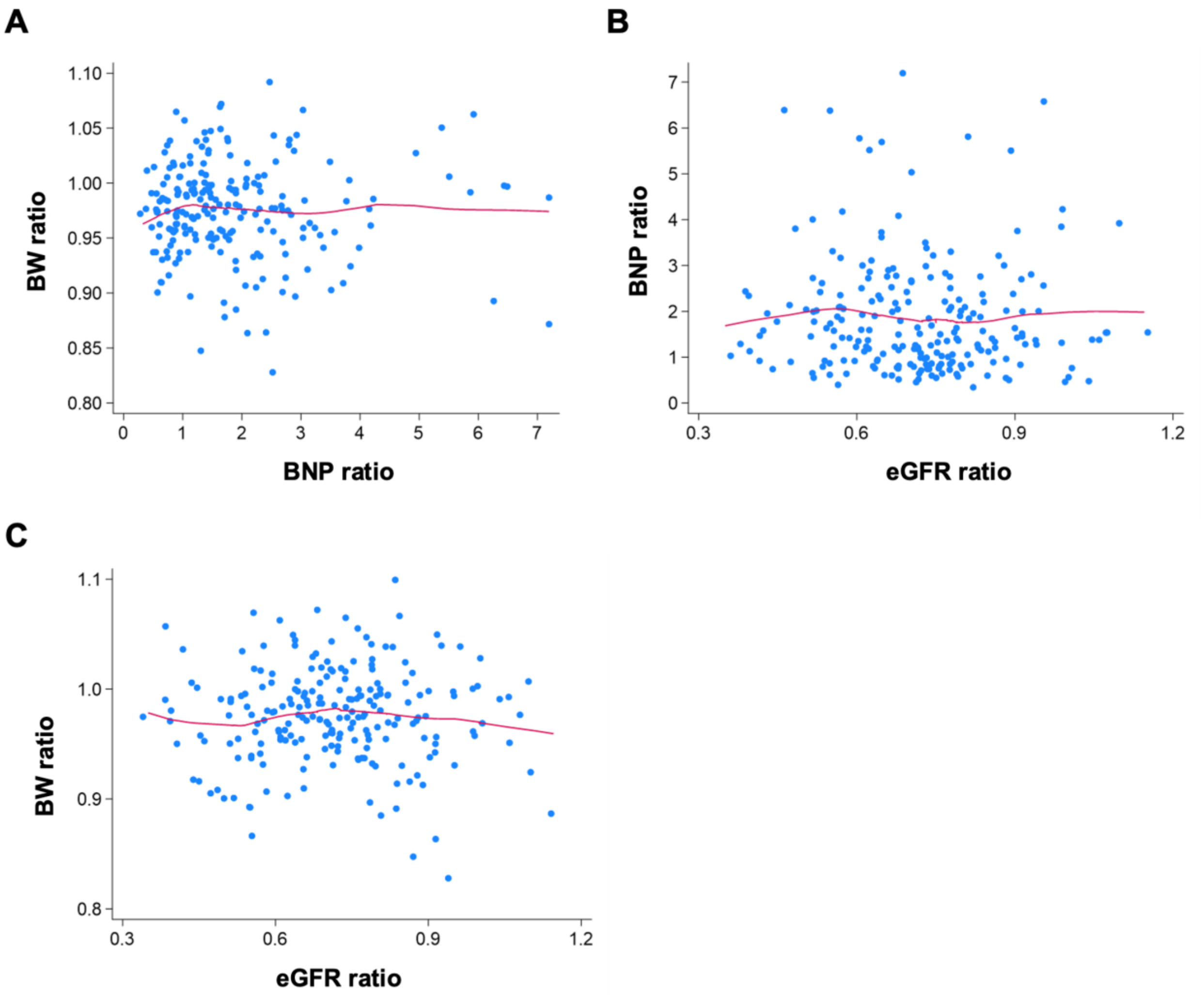

Supplement: Supplementary File (PDF) — Tables S1-S5, Figure S1. [file mmc1.pdf]
